# Identification of a novel nonsense mutation and a recurrent missense mutation in UROS gene in a patient with congenital erythropoietic porphyria 

**DOI:** 10.3389/fgene.2025.1486595

**Published:** 2025-03-31

**Authors:** Ning Jia, Yusupu Yimin, Ming Li, Long Jiang, Yeqiang Liu

**Affiliations:** ^1^ Department of central laboratory, Shanghai Skin Disease Hospital, School of Medicine, Tongji University, Shanghai, China; ^2^ Department of Dermatology, Kashi Prefecture Second People’s Hospital, Xinjiang, China; ^3^ Department of Dermatology, The Children’s Hospital of Fudan University, Shanghai, China; ^4^ Department of Dermatologic Surgery, Shanghai Skin Disease Hospital, School of Medicine, Tongji University, Shanghai, China; ^5^ Department of Pathology, Shanghai Skin Disease Hospital, School of Medicine, Tongji University, Shanghai, China

**Keywords:** uroporphyrinogen III synthase, uroporphyrinogen I, mutation detection, erythropoietic porphyria, Sanger sequencing

## Abstract

**Background:** Congenital erythropoietic porphyria (CEP, OMIM #263700) is a rare autosomal recessive disease characterized by skin photosensitivity, hypertrichosis, scarring in light-exposed areas, erythrodontia, and dark-reddish urine. The severity of the clinical phenotype is directly associated with the complete loss of enzymatic activity resulting from UROS mutations.

**Methods:** To understand the genetic etiology of CEP in a 9-year-old female proband, we checked clinical data and collected peripheral blood samples from her and her parents. Genomic DNA was isolated and subjected to polymerase chain reaction (PCR) amplification. Sanger sequencing was performed to detect potential mutations. Bioinformatics analysis was performed to assess the pathogenicity of the identified variant, and 3D protein modeling was conducted to predict its impact on protein structure.

**Results:** The proband presents with red wine-colored urine in early infancy, reddish-brown, notched incisors, and vellus hair on the forehead and trunk. Blisters develop on sun-exposed areas, leaving hyperpigmented macules after rupture. Sanger sequencing identified a previously reported missense mutation (c 0.425C > T: p.P142L) and a novel nonsense mutation in the UROS gene (c 0.325A > T: p.K109*). Bioinformatic analysis indicated that the c 0.325A > T: p.K109* variant is pathogenic. Structural modeling demonstrated that the heterozygous c.325A > T transversion in exon 6 of UROS caused a K109 termination at the protein’s α6 helix chain.

**Conclusion:** Our findings underscored the critical role of Sanger sequencing in the accurate diagnosis of atypical CEP cases and in facilitating informed genetic counseling. The identification of a UROS gene novel mutation in this case indicates a mild phenotype, further expanding the spectrum of disorders associated with UROS variants.

## Introduction

Congenital erythropoietic porphyria (CEP, OMIM #263700), also known as Gunther’s disease, is a rare autosomal recessive disorder caused by mutations in the UROS gene, leading to reduced activity of uroporphyrinogen-III-synthase (UROS) (Mirmiran et al., 2021). Clinically, CEP is characterised by skin photosensitivity, hypertrichosis and scarring on light-exposed areas, erythrodontia (reddish-brown discolouration of the teeth) and dark-reddish urine (Mathews et al., 2001). Some patients also show hemolytic anaemia, hypochromia, anisocytosis and splenomegaly due to porphyrin accumulation in erythrocytes (Moghbeli et al., 2012). Laboratory examinations typically reveal elevated levels of type-I isomer uroporphyrins and coproporphyrins in plasma, urine, and fecal samples (Stölzel et al., 2019). Diagnosis is confirmed through genetic analysis of UROS mutations and assessment of enzymatic activity (Stölzel et al., 2019). Heme biosynthesis comprises eight steps, with the fourth step involving the conversion of linear tetrapyrrole hydroxymethylbilane to cyclic tetrapyrrole uroporphyrinogen III, catalysed by the enzyme *UROS*. Mutations in the *UROS* can lead to the non-enzymatic cyclisation of hydroxymethylbilane to the I-isomer of uroporphyrinogen (URO-I), which further metabolized to coproporphyrinogen-I (COPRO-I) by uroporphyrinogen decarboxylase but cannot be converted into heme (Solis et al., 2001; Balwani and Desnick, 2012; Puy et al., 2010). Reduced *UROS* activity results in the accumulation of type I porphyrins, URO-I, and COPRO-I in various tissues (Freesemann et al., 1998; Aizencang et al., 2000). The risk of CEP is limited to severe, such as ante/prenatal clinical presentation with hydrops or severe hemolytic anaemia (Erwin and Desnick, 2019). This case study presents a novel nonsense mutation and a previously reported mutation in the UROS gene identified in a 9-year-old girl with mild CEP symptoms.

## Material and methods

Under the guidelines of the Ethics Committee of the Skin Disease Hospital Affiliated with Shanghai Tongji University School of Medicine, informed consent was obtained from the patient’s family before the DNA testing was conducted. The testing procedure follows the principles outlined in the Helsinki Declaration.

Genomic DNA was extracted from blood samples collected from the subjects and fragmented into fragments of 150–250 base pair segments using a Covaris sonicator. Following end repair and A-tailing, Y-shaped adapters were ligated to the fragments to construct DNA libraries. These libraries were labeled with distinct index tags and subsequently pooled together for further analysis. Liquid-phase hybridization was performed using biotinylated probes to capture all exons of the target genes bound to magnetic beads via streptavidin. The captured DNA was linearly amplified by PCR, and the quality of the library was assessed. High-quality libraries were subsequently sequenced on the Illumina Hi Seq X Ten platform. DNA was extracted from blood samples and fragmented into pieces measuring 150–250 base pairs using a Covaris sonicator. Following the repair and addition of A-tails, these fragments were ligated to Y-shaped adapters to generate DNA libraries. Distinct index tags were employed to distinguish between the libraries, which were later pooled together. Then, Liquid-phase hybridization was carried out with biotinylated probes to capture all exons of the target genes onto magnetic beads via streptavidin. The captured DNA underwent linear amplification via PCR, followed by evaluation of the library quality. Only libraries meeting high-quality standards were then subjected to sequencing on the Illumina HiSeq X Ten platform. This study utilized a probe panel that covers over 500 genes closely associated with genetic skin disorders. The Illumina Hi Seq X Ten sequencing platform was selected, achieving a remarkable average sequencing depth of over 200X and an outstanding Q30 score exceeding 90%.

The raw data from the offline sequencer is processed using Illumina CASAVA 1.8 to convert the BCL files into FASTQ files. The read sequences are aligned to the human reference genome (GRCh37/hg19) by BWA, Sam tools, and Picard software. Following alignment, mutation detection is performed using the GATK software suite, which includes steps like realignment and duplicate removal. The resulting variant calling files (VCF) are annotated with ANNOVAR and filtered based on parameters such as frequency, genomic position, functional impact, genetic mode, and clinically relevant phenotypes. Sanger sequencing was utilized to validate the parental relationship by employing reaction systems consisting of dideoxy nucleotide triphosphates, DNA polymerase, target fragments, and sequencing primers. The reaction products were separated and analyzed using gel electrophoresis and X-ray gel autoradiography. The template fragment guided DNA replication initiated by the primer-bound DNA polymerase, with the reaction terminating upon encountering dideoxy nucleotide triphosphates. Subsequently, electrophoretic separation of the reaction products was conducted on a 1.5% agarose gel, followed by examination using X-ray gel autoradiography. Computer analysis identified longitudinal bands displaying consistent patterns as electrical signals, with data obtained through DNA sequencing analysis technology.

## Results

### Case report

**FIGURE 1:**
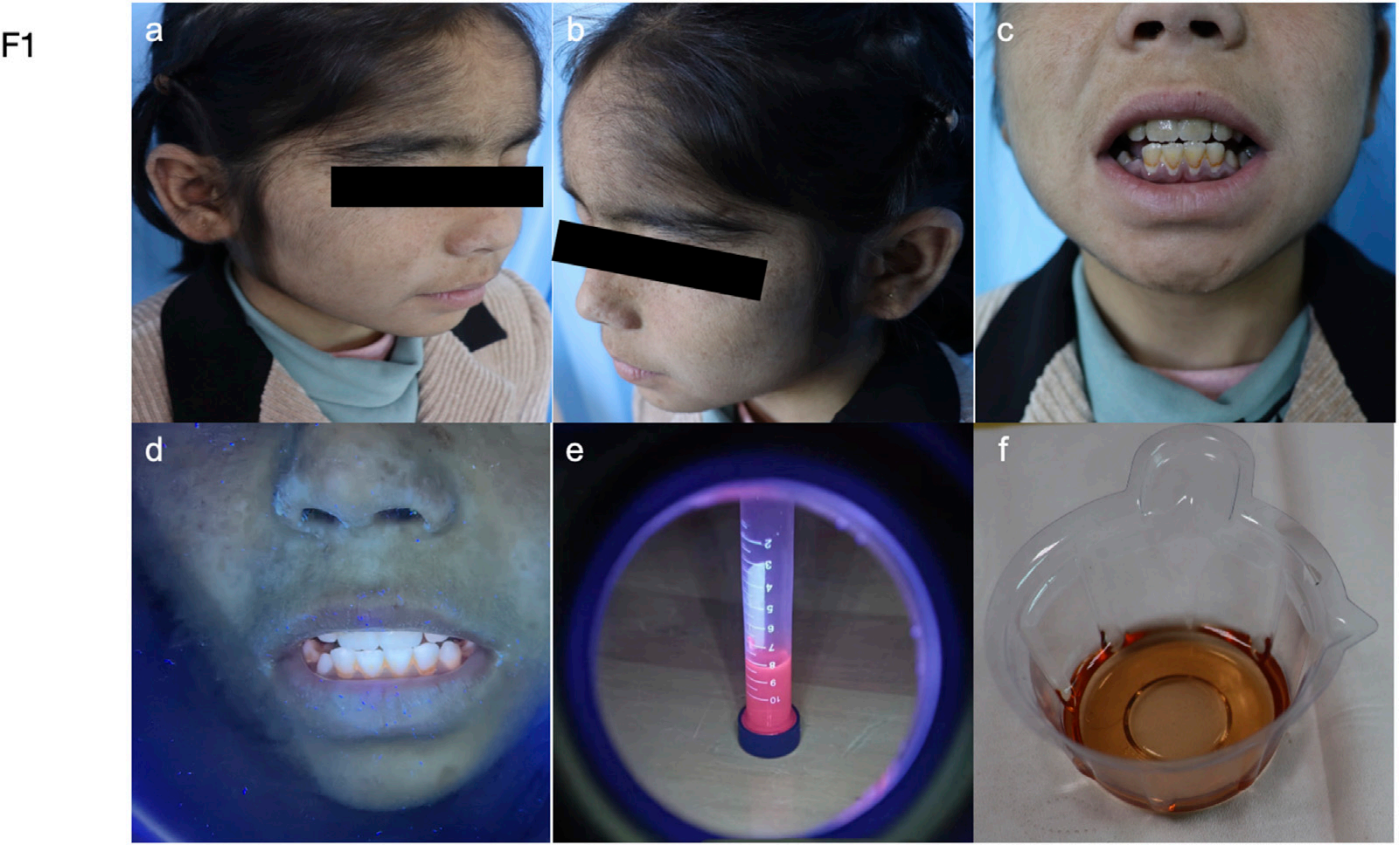
Manifestations of the patient. **(a–b)** Hypertrichosis on the forehead, scaling, and hyperpigmented patches on the cheeks. **(c–f)** Photographs show that the patient’s teeth and urine are brownish-red and fluoresce under the ultraviolet light of a Woods lamp.

In 2023, a 9-year-old girl was referred to our hospital in 2023 with worsened skin lesions of congenital erythropoietic porphyria (Figure 1). She had vellus hair on her forehead, atrophic scars scattered on the face with hyperpigmented patches densely distributed on these scars, depigmented macules, and atrophic scars scattered on the hand (Figure 1). Upon illumination with Wood’s lamp, erythrondontia and red urine were evident (Figure 1). There were no significant changes in haematological parameters, thrombocytopenia, or anaemia. However, some hepatic and renal biochemical parameters were abnormal. Alkaline Phosphatase and adenosine deaminase levels were raised to 208.20 U/L and 38.5 U/L, respectively (reference range: 35–100 U/L and 0–20 U/L). Creatinine levels were declined to 28.02 umol/L (reference range: 45–104 umol/L), and ɑ1-microglobulin levels were decreased at to 6umol/L (reference range: 10–30 umol/L). Urine analysis revealed the presence of bilirubin (+/−), L-urobilinogen (1+), and urobilinogen (1+). The patient’s hematological parameters, including red blood cell count, hemoglobin levels, and platelet indices, were all within normal limits, showing no significant abnormalities.

The child presented with red wine-coloured urine in the first 6 months of life. Upon eruption of her first teeth, her parents observed that they were reddish-brown with notched incisor tips. Vellus hair was present on her forehead and trunk. Blisters appeared on sun-exposed areas such as the face and dorsum of the hand. Hyperpigmented macules were observed on skin that had been damaged due to blister rupture. Despite this presentation, doctors at the nearby county hospital could not make a conclusive diagnosis. At the age of 5, she was diagnosed with congenital erythropoietic porphyria at the People’s Hospital of Xinjiang Uygur Autonomous Region. Treatment with hydroxychloroquine and β-carotene resulted in smaller and fewer blisters on her face and hands. However, the patient discontinued therapy for personal reasons, and the blisters on her face and the skin of her hands increased in size, ruptured, and became more numerous. By age 7, she showed no improvement after seeking care at the First People’s Hospital of Kashgar Region. The patient had no previous history of liver disease or tuberculosis, and there was no family history of similar dermatologic disorders. The patient has consanguineous parents.

### Genetic analyses

**FIGURE 2:**
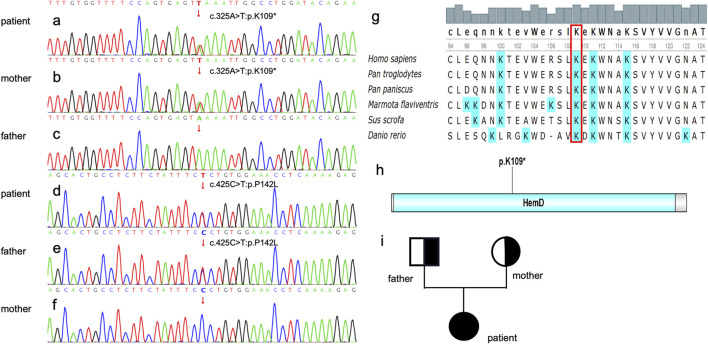
cDNA verification. **(a)** The patient carries the heterozygous mutation of c. 325A>T. **(b)** The patient’s mother carries c. 325A>T. **(c)** The mutation c. 325A>T was not found in the proband’s father’s blood sample. **(d)**The patient carries the heterozygous mutation of c. 425C>T. **(e)** The patient’s father carries c. 425C>T. **(f)** The c. 425C>T mutation was not found in the proband’s mother’s blood sample. Genetic Conservativeness Analysis and Protein structural domain analysis **(g)** The K109 locus is highly conserved in several species of *Homo sapiens*, *Pan troglodytes*, *Pan paniscus*, Marmota flavivirids, *Sus scrofa* and *Danio rerio*. **(h)** K 10 9 is located within the Hem D, a putative conserved domain for UROS. **(i)** pedigree chart, father and mother are carriers of a genetic mutation, and their daughter is affected.

**FIGURE 3:**
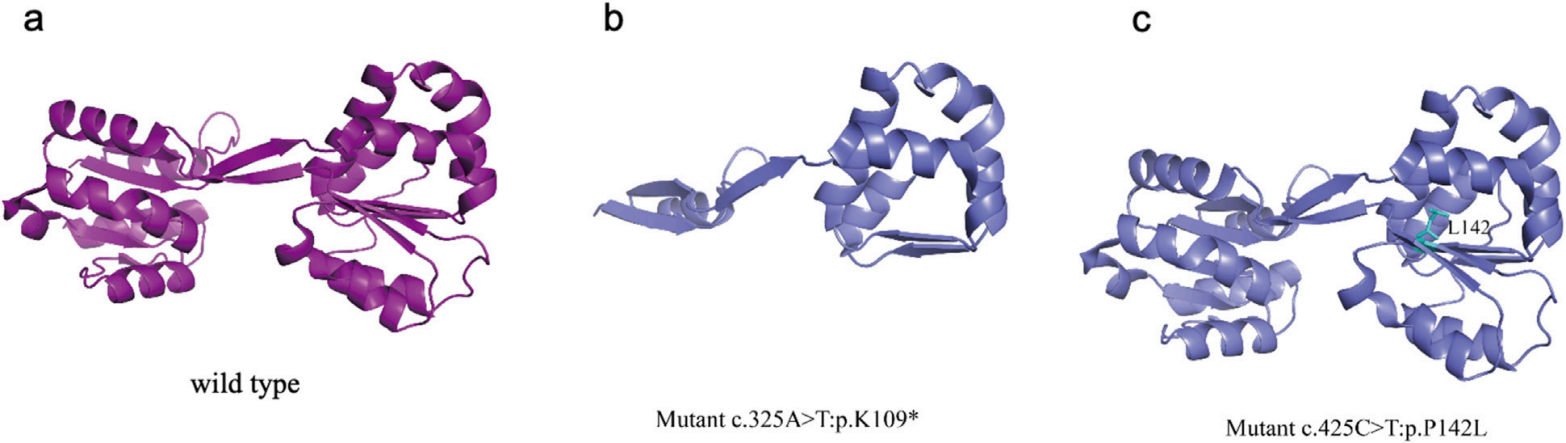
*UROS* wild-type and mutated protein structures **(a)** *UROS* wild-type structure **(b)** The mutations p.K109*, identified in the patient, K109 is highly conserved among the *UROS* protein **(c)** The mutations p. P142L, identified in the patient, resulting in the local unfolding of the protein.

Genetic analysis revealed that the patient had a heterozygous nonsense mutation (c.325A>T, p.K109*) and a heterozygous missense mutation (c.425C>T, p.P142L) (Figures 2a, d). The mother was identified as a carrier of the heterozygous nonsense mutation c.325A>T (p.K109*), as shown in Figure 2b. No mutation at this locus was detected in the father (Figure 2c). In contrast, the father was identified as a carrier of the heterozygous missense mutation c.425C>T (p.P142L), as illustrated in Figure 2e. The mother, however, did not harbor the mutation at this locus (Figure 2f). Parental verification confirmed that the patient inherited the two pathogenic variants from her father and mother, respectively (Figure 2i). The variant c. 325A>T: p. K109 * is in the conserved domain (Shaik et al., 2012), and most of the proteins translated by c.425C>T: p.P142L are within the conserved domain (Figures 2g, h), which indicates both of the two variants are pathogenic (Figure 3). c.325A>T (p. Lys109*) in the *UROS* gene [NM_000375.3] - classification as - likely pathogenic (PVS1, PM2 criteria); (ii) variant c.425C>T (p. Pro142Leu) in the *UROS* gene [NM_000375.3] - classification as - VUS (PM2 and PP3 criteria).

The *UROS* enzyme is composed of two identical subunits, each containing eight α-helices and four β-strands. The crystal structure illustrates the formation of a large antiparallel β-sheet interaction domain between the *UROS* subunits, which likely contributes to their tight assembly and interaction (Mathews et al., 2001). This interaction domain potentially improves the catalytic efficiency and stability of *UROS*. Furthermore, the crystal structure of *UROS* reveals two nested binding sites for products, which may be involved in substrate binding, product release, and interactions with other molecules that regulate UROS catalytic activity (Mathews et al., 2001).

The mutations at c.425C>T: p.P142L, which was identified in the patient, have been reported by another group (Weiss et al., 2019). This mutation resulted in a limited loss of interresidue contacts (with residues 142) and caused a slight oscillation in the open inter-domain distance, leading to localized protein unfolding (Figure 3). The mutation p.K109*, identified in the patient, was novel and have not been reported in other studies. The heterozygous c.325A>T transversion in exon 6 of UROS caused a K109 termination at the protein’s α6 helix chain. K109 is highly conserved among the *UROS* protein from five mammalian species (Mathews et al., 2001). Previous work suggested that this mutation could disrupt the packing of the adjacent conserved residue I110 in domain 1 due to the presence of a premature termination codon (Mathews et al., 2001). As a result, disrupted tertiary structures tend to make proteins unstable, less soluble, or quicker to degrade, leading to disease symptoms.

## Discussion

Congenital erythropoietic porphyria (CEP) is a rare inherited metabolic disorder caused by mutations in the *UROS* gene, in which lead to deficient activity of the enzyme uroporphyrinogen III synthase activity (Erwin and Desnick, 2019). CEP typically presents with late-onset cutaneous porphyria. Symptoms include itching, blisters, oedema, and hyperpigmentation (Aguilera et al., 2016). Hemolytic anaemia is often observed due to porphyrin accumulation in red blood cells (Blouin et al., 2017). Secondary clinical manifestations may include dental abnormalities, bone deformities, ocular complications, and neurological issues (Erwin and Desnick, 2019). CEP follows an autosomal recessive inheritance pattern affecting individuals from various ethnic backgrounds (Erwin and Desnick, 2019). The prognosis of CEP varies based on symptom severity and complications. Early diagnosis and appropriate management are essential for improving the quality of life of affected individuals (Erwin and Desnick, 2019). Treatment may include sun and UV exposure prevention, blood transfusions, hematopoietic stem cell transplantation, and investigational therapies such as gene therapy or enzyme replacement (Erwin and Desnick, 2019).

Up to July 2018, the Human Gene Mutation Database (HGMD) documented 51 pathogenic UROS mutations. The majority were missense mutations (59%, n = 30), followed by regulatory mutations (12%, n = 6) and splice site mutations (8%, n = 4) (Stölzel et al., 2019). Rare nonsense mutations, small and large insertions and deletions, and complex rearrangements have been reported. Notably, the p.C73R mutation in *UROS* is the most prevalent, with less than 1% of enzyme activity compared to the wild-type allele (Xu et al., 1995). Upon examining the correlation between genotype and clinical symptoms, it has become evident that patients with mutations leading to complete loss or substantial reduction in enzyme activity tend to present more severe clinical manifestations, including progressive hemolytic anaemia, splenomegaly, and osteoporosis. In contrast, patients with mutations show higher residual activities, such as p.A104V (7.7% of normal activity), generally present with milder symptoms of CEP (Desnick and Astrin, 2002; Shady et al., 2002). Another patient, who carried a heterozygous mutation (p.C73R) and a promoter mutation with increased activity (−76A), showed a mild form of cutaneous disease (Desnick and Astrin, 2002). All mutations discussed in this study were heterozygous, which may partially explain the moderate severity of CEP observed in this patient.

In this study, the proband carries a nonsense mutation (c.325A>T, p.K109*). This mutation has not been reported in CEP studies. Both Tanigawa et al. and Xu et al. identified a nonsense mutation (c.745C>T, p.Q249X) in the UROS gene (Tanigawa et al., 1996; Xu et al., 1995). Tanigawa et al. demonstrated that the mutated protein, expressed heterologously in *E. coli*, showed no residual enzymatic activity. Xu et al. reported that despite the second allele lacking detectable activity, patients had milder clinical manifestations (Tanigawa et al., 1996; Xu et al., 1995). In our research,we found a novel nonsense mutation (c.325A>T, p.K109*), resulting in the deletion of exon 6, spanning 97 bases in the mRNA, predicted generate protein truncation based on bioinformatics analysis (Figure 3B). We speculated that enzyme activity could be affected and despite the different mutation status in *UROS*, the patient’s mild clinical presentation consistent with reported cases with nonsense mutations. On the other hand, the proband inherited a missense mutation (c 0.425C>T: p.P142L) from her father. This mutation has been previously reported in American patients with CEP (Weiss et al., 2019). Yedidyah et al. found that 31% of unrelated individuals with CEP had *UROS* missense mutations in CEP, including c.425C>T: p.P142L (Weiss Y et al., 2019). Missense mutations mainly cause disease by impairing protein folding and stability, resulting in thermodynamic instability, premature degradation, and disruption of cellular protein homeostasis (Blouin et al., 2017). The severity of the condition is associated with the pathways of structural destabilisation (Blouin et al., 2017). Clinical phenotypes were influenced by factors such as the environment, photoprotective behaviour, and genes beyond *UROS* (Katugampola et al., 2012). The clinical manifestations resulting from a truncated or abnormal UROS protein are generally less severe than those caused by a complete deletion of the CEP protein. Additionally, homozygous mutations may lead to a more severe phenotype (Xu et al., 1995). In our study, the parents of the patient carried pathogenic *UROS* mutations without any clinical symptoms, while the patient with both mutations presented with CEP symptoms. This interesting finding differs from previous reports. CEP is a complex systemic disease. In addition to hematological complications, manifestations affecting the skin, eyes, oral cavity, and skeletal system contribute to increased disease severity and reduced health-related quality of life. The illness’s rarity often leads to diagnosis delays (Katugampola et al., 2012). Early detection, diagnosis, and intervention, particularly with haematological monitoring, are essential for improving patients' quality of life.

At birth, the proband displayed hypertrichosis, skin photosensitivity, erythrodontia, and dark-reddish urine, indicating a likely mild severity of the phenotype and manifestation of CEP (Moghbeli et al., 2012). Patients with mutations expressing higher residual activities, such as the p.A104V mutation (7.7% of regular activity), typically present with moderate or mild forms of CEP (Desnick and Astrin, 2002). In addition to residual enzyme activity, the manifestation of the CEP phenotype is also potentially influenced by variants in other genes of the heme biosynthesis pathway (Balwani et al., 2013; Bishop et al., 2013; Whatley et al., 2008). A precise diagnosis of CEP is crucial for providing accurate prognosis guidance, genetic counselling, and prenatal diagnosis (Stölzel et al., 2019). The patient’s *UROS* genotype is predictive of disease severity, especially when correlated with *in vitro* mutation studies. Without allogeneic hematopoietic stem cell transplantation, disease management focuses on avoiding sun or light exposure and vitamin D supplementation (Stölzel et al., 2019).

In conclusion, a novel nonsense mutation was identified in a proband who displayed mild CEP manifestations. The analysis of mutations in genes responsible for CEP is essential for effective patient management. Genetic counselling for the family, along with prenatal diagnosis, is highly recommended for such disorders. In regions with a high incidence of consanguineous marriages, it is essential to provide thorough genetic counselling to affected families. This study highlights significant phenotypic variability and differences in disease severity that can occur among individuals with identical genotypes.

## Data Availability

The datasets presented in this study can be found in online repositories. The names of the repository/repositories and accession number(s) can be found in the article/supplementary material.
